# Value of Examination of the Mouth in the Choice of of a Nurse

**Published:** 1898-03

**Authors:** V. Jarre


ARTICLE III.

### VALUE OF EXAMINATION OF THE MOUTH IN THE CHOICE OF A NURSE.

BY DR. V. JARRE.

Reported by George Randolph, Berlin, Germany.

Doctors and midwives are usually guided in their choice of a wet-nurse by several general considerations, amongst which there is one, the value of which has hitherto not been sufficiently appreciated, viz., the examination of the mouth.

Mauricean, who is even to day an authority on such questions, demands a nurse whose mouth is absolutely healthy; whose teeth must be white and sound without any caries, because in kissing the child constantly, she would infect its lungs by making it breathe her foul breath. Every wet-nurse ought to present herself with a mouth in perfect condition.

But it is well known that the dental caries is very frequent in our white race, and there are in France for example, vast regions where the complaint is so common that one very rarely finds a young woman with all her teeth sound.

The presence of one or more carious teeth does not constitute an immediate danger of inflammatory and septic complications. However, such teeth may be the seat of painful sensations, produced either by the contact of hot or cold food, or by the pressure of some hard food entering the cavity, during mastication. Such accidents may bring on a more or less pronounced functional disturbance, affecting the nurse's milk. Moreover it is well known that the period of nursing, as well as that of pregnancy, prediaposes the advance of caries in quite a peculiar manner.

The conclusion to be drawn from these considerations, is that treatment of all cases, followed by filling of the cavities, ought to be imposed upon every wet-nurse, who suffers from dental lesions either at the beginning or during the course of nursing.—*Items of Interest.*

